# Diagnosis of acute canine leptospirosis using multiple laboratory tests and characterization of the isolated strains

**DOI:** 10.1186/s12917-018-1547-4

**Published:** 2018-07-17

**Authors:** Bruno Alonso Miotto, Barbara Furlan Tozzi, Manoela de Souza Penteado, Aline Gil Alves Guilloux, Luisa Zanolli Moreno, Marcos Bryan Heinemann, Andrea Micke Moreno, Walter Lilenbaum, Mitika Kuribayashi Hagiwara

**Affiliations:** 1 Departamento de Clínica Médica (Department of Veterinary Clinics), Faculdade de Medicina Veterinária e Zootecnia (School of Veterinary Medicine and Animal Science), Universidade de São Paulo (University of São Paulo), São Paulo, SP 05508-270 Brazil; 2 Departamento de Medicina Veterinária Preventiva e Saúde Animal (Department of Veterinary Preventive Medicine and Animal Health), Faculdade de Medicina Veterinária e Zootecnia (School of Veterinary Medicine and Animal Science), Universidade de São Paulo (University of São Paulo), São Paulo, SP 05508-270 Brazil; 3 Departamento de Microbiologia e Parasitologia (Department of Microbiology and Parasitology), Universidade Federal Fluminense, Niterói, RJ 24210-130 Brazil

**Keywords:** Leptospirosis, Dogs, Canine, PCR, MAT, Acute infection, Culture, Sequencing, MLST, Icterohaemorrhagiae

## Abstract

**Background:**

Dogs presenting with acute leptospirosis may present non-specific clinical and laboratory findings, and the definitive diagnosis may require additional confirmatory tests, including bacterial culture, for the direct or indirect identification of the pathogen. The present study describes the diagnosis of leptospirosis in suspected dogs based on the use of multiple diagnostic tests, including serological, molecular and bacteriological tests, along with the characterization of the recovered leptospiral strains.

**Results:**

Urine, serum and blood samples were collected from 33 dogs with suspected clinical leptospirosis treated at the University of São Paulo Veterinary Hospital Service (Hovet FMVZ-USP) between 2013 and 2016. Only dogs with high blood urea nitrogen and creatinine levels in association with multiple clinical manifestations of the disease were included. Leptospiral culture, PCR and serology (Microscopic agglutination test - MAT) were performed in blood and urine samples taken from all suspected dogs at clinical presentation, and an additional prospective MAT titration was performed in seven dogs. Infection could be identified exclusively by PCR in 10 dogs (30.3%), exclusively by MAT in four dogs (12.1%) and by both tests in four dogs, totaling 18 dogs (54.5–95%CI: 37.6–71.5). Six out of eight MAT-confirmed cases presented with the highest titers against the Icterohaemorrhagiae serogroup. Leptospires were recovered from urine samples from two PCR-positive dogs, and both strains could be characterized by Multilocus Sequence Analysis and serogrouping as *L. interrogans* serogroup Icterohaemorrhagiae. Both isolates were shown to be pathogenic in the hamster model.

**Conclusions:**

The simultaneous use of MAT and PCR was able to increase the diagnosis of leptospirosis in clinically suspected cases. Despite the increasing incidence of new serovars affecting dogs being reported in different locations, our results suggest that leptospiral strains belonging to the Icterohaemorrhagiae serogroup are still a major causative agent of canine leptospirosis in São Paulo, Brazil.

**Electronic supplementary material:**

The online version of this article (10.1186/s12917-018-1547-4) contains supplementary material, which is available to authorized users.

## Background

Leptospirosis is a bacterial disease caused by infection with pathogenic species of the genus *Leptospira,* which can affect virtually all mammals [1]. Dogs are considered highly susceptible to the infection because of a marked environmental exposure to leptospires, and canine leptospirosis has been largely described worldwide [1, 2]. Recent reports have shown the reemergence of clinical illness in dogs and humans [1, 3], highlighting the importance of improving current diagnostic approaches and prevention strategies.

Infected dogs may manifest a broad spectrum of clinical symptoms, varying from hepatic and renal failure, often accompanied by hemorrhagic and pulmonary disorders, to mild, self-limiting febrile illness and asymptomatic infections [4]. Clinical and laboratory findings are usually non-specific, and a definitive diagnosis requires additional confirmatory tests for the direct or indirect identification of the pathogen, such as dark-field microscopy, Polymerase Chain Reaction (PCR), bacterial culture and Microscopic agglutination test (MAT) [1].

Since the exact time of infection at clinical presentation is typically unknown, and given that the early and accurate identification of infected dogs is crucial to alter the course of the disease with appropriate drug therapy [5], the use of multiple simultaneous tests may improve the chance for a correct diagnosis and consequent therapeutic success [6]. Despite the development of several serological tests for the detection of anti-*Leptospira* antibodies in recent years [7–9], the MAT is still widely employed for the serodiagnosis of acute leptospiral infection [10]. However, critical limitations may hamper its use in a clinical setting; the evaluation of one single MAT test may fail to detect antibodies at the early phase of the disease, and titration of convalescent serum samples is frequently required to reveal seroconversion, which may impose some diagnostic difficulties due to the high mortality of the disease. MAT also has a poor ability to predict the infecting serovar and may not distinguish between infection and vaccine-induced titers [10].

Conversely, PCR has been successfully used to confirm leptospiral infection at the early stages of infection [6, 11], and sequencing PCR amplification products has enabled the identification of the different leptospiral species infecting dogs [12, 13]. Nonetheless, culturing leptospires still stands as the gold standard reference test to unmistakably confirm leptospiral infection, and only serological characterization of the isolated strains may provide reliable information regarding serogroup or serovar identity [1]. The establishment of a panel of leptospiral strains circulating among dog populations remains the major strategy to support the development and commercialization of vaccines with more specific serovar composition, which would hypothetically increase immunization effectiveness for local canine populations. Unfortunately, culture is challenging due to frequent contamination and the fastidious growth of the pathogen [1]. Recovering leptospires from suspected dogs is also limited by the early institution of therapeutic intervention, which is usually required after the disease is suspected [14].

Few reports have studied canine leptospirosis in Brazil [15], and the characterization of leptospiral strains recovered from suspected clinical cases is poorly documented [16]. Given the benefits of using multiple laboratory tests to increase the likelihood of an accurate diagnosis, we hereby describe the diagnosis of leptospirosis in suspected cases treated at a University reference center, based on the association of multiple diagnostic strategies, such as serological, molecular and bacteriological tests, along with the characterization of the recovered leptospiral strains.

## Methods

### Sample collection and inclusion criteria

Urine and blood samples were collected from 33 dogs with suspected clinical leptospirosis presented to the University of São Paulo Veterinary Hospital Service (Hovet FMVZ-USP) between 2013 and 2016. The dogs were suspected of acute leptospirosis when presenting with high serum blood urea nitrogen (BUN) and creatinine levels of unknown origin (> 60 mg/dL and 1.4 mg/dL, respectively) in association with two or more clinical manifestations suggestive of leptospirosis (hemorrhagic disorders, fever, vomiting, jaundice, prostration, hyporexia/anorexia). Dogs presenting with polyuria, polydipsia and weight loss a month prior to the presentation at the veterinary ambulatory were not included in the study in order to rule out chronic kidney disease bias.

Leptospiral culturing, PCR and MAT were performed in all suspected dogs at clinical presentation, and samples were collected prior to the institution of antimicrobial therapy. Biochemistry and hematological parameters were also determined and included blood urea nitrogen (BUN) and creatinine (CR) serum concentrations, alkaline phosphatase (ALP) and alanine aminotransferase activity (ALT), including also hematocrit (Ht) and white blood cell count (WBC). Reevaluations were performed in seven dogs and included exclusively serum antibody titration and evaluation of biochemistry and hematological parameters; no PCR or bacterial culture was performed in the prospective evaluation.

Blood samples were collected from the jugular or cephalic vein and drawn into BD Vacutainer tubes (BD Diagnostics, New Jersey, USA) and Venosafe™ tubes containing K_3_ EDTA (Terumo, Terumo Europe N.V, Leuven, Belgium) to obtain serum and whole-blood samples, respectively. All urine samples were taken aseptically by cystocentesis.

### Diagnostic criteria

Acute leptospiral infection was confirmed by the detection of at least a four-fold increase in MAT titers between acute and convalescent serum samples [1] and/or the presence of MAT titers ≥800 in single serum samples from non-vaccinated dogs, as previously suggested [5]. Dogs with positive PCR results (urine and/or blood samples) were considered to be infected only after identification of *Leptospira* spp. was confirmed by DNA sequencing.

### Anti-*Leptospira* antibody detection

MAT was performed to detect anti-*Leptospira* antibodies in patient’s serum samples [17] using a panel of 22 serovars representing 18 serogroups, as previously described [12]. Endpoint titers were determined using two-fold dilutions until the last well showing 50% agglutination was recorded. The cut-off for a positive MAT reaction was defined as a titer ≥100; however, only non-vaccinated dogs with single MAT titers of 800 or above were considered to be acutely infected.

### Culturing of *Leptospira*

For the recovery of leptospires, 0.5 mL aliquots from blood and urine samples were diluted in sterile physiological solution to a final concentration of 1:10 and 1:100, and 0.5 mL of each solution was further inoculated in semi-solid Fletcher and liquid EMJH medium (Difco Laboratories, Franklin Lakes, NJ, USA). The tubes were incubated at 28 °C for 12 weeks and examined weekly by dark-field microscopy for the presence of spirochetes.

### Leptospiral DNA detection

DNA was extracted from blood or urine samples using NucliSens® miniMAG™ (BioMérieux Inc., Durham, NC, USA) according to the manufacturer’s instructions. Urine samples were centrifuged (6.500 G-force, 25 °C, 25 min) and pellets were resuspended in 1 ml sterile phosphate-buffered saline (PBS – pH 7.2) prior to DNA extraction. Extracted DNA was subjected to PCR amplification using a previously reported *Leptospira* genus-specific protocol and primers targeting a 331 bp fragment of the 16S rRNA gene [18]. Cycling conditions were carried out as follows: 94 °C for 5 min, 40 cycles at 94 °C for 30 s, 60 °C for 30 s, 72 °C for 30 s and a final extension at 72 °C for 5 min. Pure *L. interrogans* serovar Canicola (strain Hond Utrecht IV) genomic DNA was used as a positive control and DNase-free water as a negative control in all PCR runs. The amplified products were separated by electrophoresis on a 2% agarose gel stained with SYBR Safe DNA gel stain (Invitrogen, Thermo Fisher Scientific Inc., Carlsbad, CA, USA) and analyzed under UV transillumination.

### 16S rRNA sequencing

The amplicons were sequenced on an ABI 7500 Genetic Analyzer (Life Technologies, Waltham, MA, USA). The sequences were edited using BIOEDIT Sequence Alignment Editor 7.0.9 (Hall, 1999 - Ibis Biosciences, Carlsbad, CA, USA) and compared to reference sequences deposited in GenBank using the BLAST tool (http://www.ncbi.nlm.nih.gov/BLAST/).

### Characterization of dog isolates

#### Serogrouping

The serogroups of the isolates were determined by MAT using a panel of rabbit anti-sera for 34 serovars representing 28 serogroups (Andamana, Australis, Autumnalis, Ballum, Bataviae, Canicola, Calledoni, Codice, Cynopteri, Djasiman, Grippotyphosa, Hebdomadis, Holland, Icterohaemorrhagiae, Javanica, Lousiana, Lyme, Manhao, Mini, Panama, Pomona, Pyrogenes, Ranarum, Sarmin, Sejroe, Seramanga, Shermani, and Tarassovi). High rates of agglutination with a particular antiserum were used to identify the presumptive serogroup of the strain [19].

#### MLST

Multilocus Sequence Typing (MLST) of the isolates using seven distinct loci (*pntA*, *sucA*, *mreA*, *glmU*, *caiB*, *tpiA*, and *pfkB*) was performed as previously described [20]. The concatenated loci were compared to *Leptospira* sequence types (STs) available in the PubMLST database (https://pubmlst.org/leptospira/) using Maximum-Likelihood method by Bionumerics 7.6 (Applied Maths NV, Sint-Martens-Latem, Belgium).

#### Virulence characterization

A pure culture of each of the isolated strains was counted in a Petroff-Hausser chamber and 0.5 mL containing 108 leptospires was inoculated intraperitoneally in thirty-day-old male hamsters (one hamster for each isolate) to determine if the isolates would produce infection [21]. The animals were purchased at Anilab Animais de Laboratório Criação e Comércio, Paulínia, SP, and were bred strictly for research purposes. The hamsters were kept in individual 30 X 20 X 12 cm polypropylene cages lined with wood shavings; free fresh water and food was daily supplied. The animals were daily monitored for signs of acute leptospiral infection (prostration, ruff hair coat, jaundice, external hemorrhage and dehydration) and were immediately euthanized after presenting two or more clinical signs. The hamster’s kidneys were aseptically removed, macerated, resuspended and inoculated in liquid EMJH medium for reisolation.

### Ethical considerations

The euthanasia procedures conducted in the animal models were in strict accordance with the recommendations in the CONCEA (National Council for Control of Animal Experimentation), and consisted of intraperitoneal administration of xylasine/ketamine and isofluran, followed by the use of a CO_2_ chamber. Dogs presenting no response to treatment were euthanized by intravenous infusion of thiopental (75 mg/kg) and acepromazine (0.05 mg/kg), followed by the intravenous use of potassium chloride (dose-response curve - average of 10 ml). All euthanasia procedures were approved by the CEUAVET Committee, as stated in the Declarations section, and all efforts were made to minimize animal suffering.

### Statistical methods

Univariate analysis was performed to describe average and standard deviation values for hematological and biochemical laboratory tests. These values were compared between leptospirosis confirmed cases (either through MAT or PCR) and non-confirmed cases using a T-test. The association of outcome with leptospirosis confirmed and non-confirmed groups was analyzed by a Chi-square test. All statistical analyses were performed in IBM SPSS Statistics 21. *P*-values of < 0.05 were considered statistically significant.

## Results

Of the 33 dogs included in the study, 18 (55.5%) had MAT titers ≥100, with titers ranging from 100 to 3200 (Table 1). The predominant reactive serogroups were Icterohaemorrhagiae (*n* = 23), Australis (*n* = 7), Pomona (*n* = 4), Butembo (*n* = 4), and Castellonis (*n* = 3). Less common serogroups included Canicola, Shermani, Cynopteri (*n* = 2), Autumnalis (*n* = 1), Pyrogenes (*n* = 1) and Sejroe (*n* = 1).

**Table 1:** Immunization records, PCR, culture, DNA sequencing and MAT results found in 33 dogs presenting clinical suspicion of leptospirosis treated at the Veterinary Hospital of University of São Paulo between 2013 and 2016

Dog	PCR		DNA sequencing	Culture	Vaccination	MAT	MAT titration (serovar)													
	Blood	Urine			(< 1 year)	(> 100)	Can	Po	Cas	Co	Ic	Py	Br	Ha	Wo	Sh	Bu	Aus	Cyn	Aut
1	NP	(+)	*L. interrogans*	(−)	(+)	(−)	–	–	–	–	–	–	–	–	–	–	–	–	–	–
2	(+)	(+)	*L. interrogans*	(−)	(−)	(+)	400	200	200	100	200	200	–	–	–	–	–	–	–	–
3	(−)	(+)	*L. interrogans*	(−)	(−)	(+)	–	800	–	3200	3200	–	200	–	–	–	–	–	–	–
4	(+)	(+)	*L. interrogans*	(−)	(−)	(+)	–	–	–	–	100	–	–	800	800	–	–	–	–	–
5	NP	(+)	*L. interrogans*	(−)	(−)	(−)	–	–	–	–	–	–	–	–	–	–	–	–	–	–
6	(−)	(−)		(−)	(+)	(+)	–	–	–	–	–	–	–	–	–	200	–	–	–	–
7	NP	(−)		(−)	(−)	(+)	–	–	–	–	–	–	–	–	–	–	–	–	100	–
8	(−)	(+)	*L. interrogans*	(+)	(−)	(+)	–	200	–	400	400	–	–	–	–	–	–	–	–	–
9	(−)	(−)		(−)	(+)	(+)	400	–	–	400	200	–	200	–	–	–	100	200	–	–
10	(+)	(−)	*L. interrogans*	(−)	(−)	(−)	–	–	–	–	–	–	–	–	–	–	–	–	–	–
11	(−)	(−)		(−)	(−)	(+)	–	–	–	800	400	–	–	–	–	–	–	–	–	–
12	(−)	(+)	*L. interrogans*	(−)	(+)	(+)	–	–	–	–	–	–	400	–	–	–	–	–	400	–
13	(−)	(−)		(−)	(−)	(−)	–	–	–	–	–	–	–	–	–	–	–	–	–	–
^a^14	(−)	(−)		(−)	(+)	(+)	–	100	–	–	–	–	–	–	–	–	–	–	–	100
^a^15	(−)	(+)	–	(−)	(−)	(−)	–	–	–	–	–	–	–	–	–	–	–	–	–	–
16	(+)	(−)	*L. interrogans*	(−)	(−)	(+)	–	–	–	400	200	–	–	–	–	–	200	–	–	–
^a^17	(+)	(+)	–	(−)	(−)	(−)	–	–	–	–	–	–	–	–	–	–	–	–	–	–
18	(−)	(−)		(−)	(+)	(−)	–	–	–	–	–	–	–	–	–	–	–	–	–	–
19	(−)	(−)		(−)	(−)	(−)	–	–	–	–	–	–	–	–	–	–	–	–	–	–
20	(−)	(+)	–	(−)	(−)	(+)	–	–	–	400	400	–	–	–	–	–	–	400	–	–
21	(−)	(−)		(−)	(−)	(−)	–	–	–	–	–	–	–	–	–	–	–	–	–	–
^a^22	(−)	(+)	*L. interrogans*	(−)	(−)	(−)	–	–	–	–	–	–	–	–	–	–	–	–	–	–
23	(−)	(−)		(−)	(+)	(+)	–	–	–	200	–	–	–	–	–	–	–	–	–	–
^a^24	(−)	(+)	*L. interrogans*	(+)	(+)	(−)	–	–	–	–	–	–	–	–	–	–	–	–	–	–
^a^25	(−)	(−)		(−)	(−)	(−)	–	–	–	–	–	–	–	–	–	–	–	–	–	–
26	(+)	(+)	–	(−)	(+)	(−)	–	–	–	–	–	–	–	–	–	–	–	–	–	–
27	(−)	(−)		(−)	(−)	(−)	–	–	–	–	–	–	–	–	–	–	–	–	–	–
28	(−)	(−)		(−)	(−)	(−)	–	–	–	–	–	–	–	–	–	–	–	–	–	–
29	(−)	(−)		(−)	(−)	(+)	–	–	100	800	400	–	–	–	–	400	–	400	–	–
^a^30	(−)	(−)		(−)	(−)	(+)	–	–	–	200	200	–	–	–	–	–	–	–	–	–
31	(−)	(+)	*L. interrogans*	(−)	(−)	(+)	–	–	800	–	–	–	–	–	–	–	–	–	–	–
32	(+)	(+)	*L. interrogans*	(−)	(−)	(+)	–	–	–	1600	–	–	–	–	–	–	200	800	–	–
33	(+)	(+)	*L. interrogans*	(−)	(−)	(+)	–	–	–	200	100	–	–	–	–	–	100	–	–	–

^a^ Dogs included in the prospective evaluation

*NP* not performed, *Can* Canicola, *Po* Pomona, *Cas* Castellonis, *Co* Copenhageni, *Ic* Icterohaemorrhagiae, *Py* Pyrogenes, *Br* Bratislava, *Ha* Hardjo, *Wo* Wollfii, *Sh* Shermani, *Bu* Butembo, *Aus* Australis, *Cyn* Cynopteri, *Aut* Autumnalis

Acute leptospirosis was diagnosed in eight dogs (24.2%) by MAT: six dogs without a history of recent vaccination had titers ≥800 in a single serum sample (dogs 3, 4, 11, 29, 31 and 32 – Table 1); additionally, prospective evaluation performed in seven dogs revealed seroconversion in two other dogs (dogs 15 and 30, see Additional file 1), which showed fourfold increase in MAT titers against Icterohaemorrhagiae serogroup (convalescent titers of 400 and 800, respectively). Overall, six dogs with MAT-confirmed leptospirosis had the highest titers against the Icterohaemorrhagiae serogroup; only one dog (dog 4) had the highest titers against the Sejroe serogroup.

Leptospiral DNA was detected in 18 out of 33 animals; however, samples from four of these dogs (dogs 15, 17, 20 and 26) did not result in readable sequences after several sequencing attempts and therefore were not considered positive, leaving 14 PCR-positive dogs (42.4% - Table 1). Positive yields were obtained from urine (*n* = 8), blood (*n* = 2) or both (*n* = 4). The 16S rRNA phylogenetic analysis showed that the recovered sequences from all PCR-positive dogs were identified as *L. interrogans* (Table 1), with high similarity (> 99%) to *L. interrogans* representatives (AY996798, AY996800). The recovered sequences were submitted to GenBank under accession numbers KX891325 - KX891333, MG640117 and MG640121.

The comparison between results from MAT-confirmed cases and PCR-positive dogs revealed that the infection could be identified exclusively by PCR in 10 dogs (30.3%; 14.6–46.0), exclusively by MAT in four dogs (12.1%; 1.0–23.3; two seroconversions and two single samples) and by both tests in four dogs. The combined use of MAT and PCR was able to detect leptospiral infection in 18 dogs, with an overall prevalence of 54.5% (95%CI: 37.6–71.5).

Clinical manifestations, laboratory findings and clinical outcome of the all dogs included in the study are presented in the Additional file 2. Clinical manifestations at presentation included vomiting (76%), hyporexia/anorexia (97%), diarrhea (33%), dehydration (33%) jaundice (57%), bleeding disorders (21%), oligodipsia, dysuria, anuria and oliguria (27%), among other symptoms. Laboratory analyses revealed average BUN and CR levels of 427.54 mg/dL (SD 186.13) and 6.96 mg/dL (SD 4.99), respectively; ALT and ALP serum activity of 165.69 IU/L (SD 240.58) and 410.78 IU/L (454.9), respectively, 38.5% Ht count (SD 10.70) and 21,954 WBC/μl (SD 12.275). Clinical outcome of 20 dogs could be determined: 10 dogs survived after proper treatment and euthanasia/death occurred in 10 cases. Thirteen dogs did not show up for follow-up care and information regarding the clinical outcome could not be determined. The comparison between dogs with confirmed infection (*n* = 18) and symptomatic dogs with no confirmation of leptospiral infection (*n* = 15) revealed no significant differences (*p* > 0.05) in BUN/CR levels, ALT and ALP activity, Ht and WBC count or clinical outcome.

Leptospires were recovered from the urine samples of two dogs (dog 8, strain DU84; dog 24, strain DU100), which had PCR-positive results: only dog 8 had titers detectable by MAT, but serum titers were found to be below 800 (Table 1). Culturing as a single diagnostic strategy had a prevalence of 6.1% (0–14.2) and had no effect in the overall prevalence when combining all confirmatory tests (PCR, MAT and culturing) for the diagnosis of leptospirosis.

**Fig. 1:**
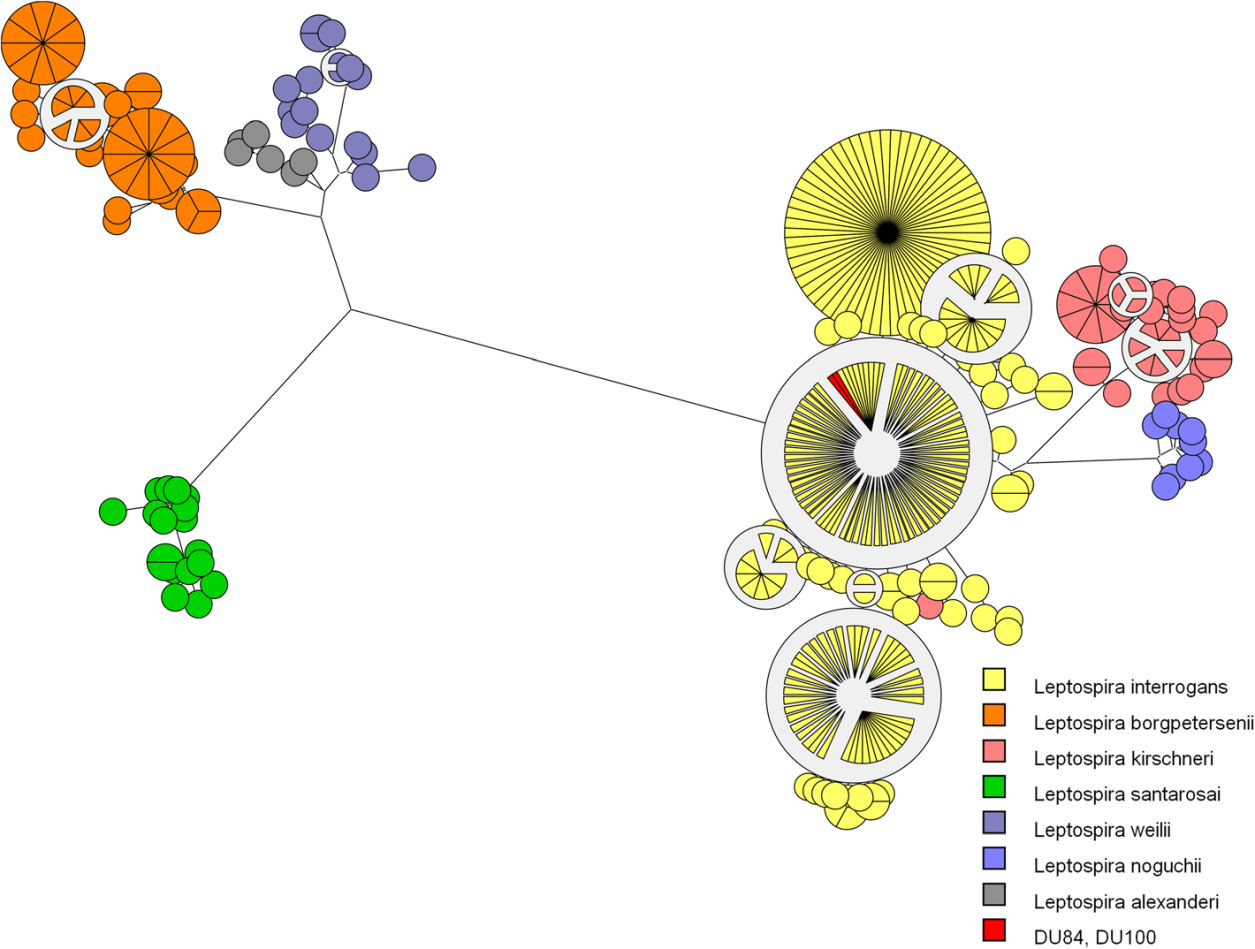
MLST analysis of the DU84 and DU100 strains. The Maximum-likelihood tree was based on the concatenated sequences of the seven loci for the 229 available *Leptospira* STs

The MLST analysis of both isolated strains (DU84 and DU100) revealed ST 17 (Fig. 1), which characterizes *L. interrogans* serogroup Icterohaemorrhagiae according to a previously described protocol [20]. Serogrouping of the DU84 strain showed a strong and specific reaction against serovars Copenhageni (6400) and Icterohaemorrhagiae (12800); the DU100 strain also had specific reactions against these serovars (Copenhageni 12,800; Icterohaemorrhagiae 51,200), revealing that both isolated strains belong to the Icterohaemorrhagiae serogroup.

The inoculation of the isolates in the hamster model produced acute and intense clinical manifestations of leptospirosis. The hamsters developed acute lethal infection within six days post-inoculation, and macroscopic alterations included epistaxis, generalized petechial stains, pulmonary/liver congestion and pulmonary hemorrhage. Leptospires were successfully recovered from the kidney and liver tissues of both hamsters after euthanasia, thus indicating leptospiral infection as the cause of disease.

## Discussion

The use of MAT and PCR (with further DNA sequencing) as independent diagnostic strategies enabled the identification of eight (24.2%) and 14 (42.4%) dogs with leptospiral infection, respectively, while the association of both tests increased the diagnosis of leptospirosis in clinically suspected cases, thus confirming the infection in 18 (54.5%) out of the 33 suspected dogs included in the study.

Although PCR was able to identify a higher proportion of infected dogs than MAT, the high number of dogs that follow-up care was discontinued and the high mortality/euthanasia rates found in the studied population has limited a more appropriate comparison between PCR and MAT results. It is usually recommended that the diagnosis of acute infection by MAT should be based on testing paired samples, collected 7 to 14 days apart, in order to detect at least a fourfold increase in agglutinating antibodies against *Leptospira* [1]. Only 7 out of 33 dogs could be prospectively evaluated, and serum samples with more than 7-day interval between acute and convalescent phases were obtained only from five dogs, including both dogs in which seroconversion was detected. Moreover, the cut off value of 800 adopted in the present study, which has been previously suggested to increase the specificity of MAT [5], and the natural delay to produce an adaptive serologic response after infection, may have led to false-negative results when testing a single serum sample by MAT. Even in face of such limitations, active leptospiral infection was determined in four dogs exclusively by MAT, while PCR was able to detect 10 infected dogs at clinical presentation that a single MAT sample evaluation could not, reinforcing that the use of multiple tests is beneficial for improving the diagnosis of canine leptospirosis in practical situations in which the clinical outcome and the possibility of follow-up care are uncertain [6].

These results are markedly different from those reported by Fraune et al. [5], who found no significant benefit in the use of PCR in relation to MAT for the early diagnosis of acute canine leptospirosis. On the other hand, our results are in agreement with the findings reported by Harkin et al. [6, 11], who found a sensitivity of 100% and specificity of 88.3% when testing a leptospiral-specific PCR assay with urine samples from dogs with clinical suspicion of leptospirosis. It is important to emphasize that the application of PCR in a clinical setting, notably when using urine specimens, may provide false-positive results [22], and the association with DNA sequencing, as performed in the present study, is highly recommended to provide more reliable results for proper comparison with other diagnostic techniques. Most dogs diagnosed by PCR and DNA sequencing were identified using urine samples, and even though the detection of leptospiral DNA in urine specimens taken from dogs with multiple signs of leptospirosis is highly suggestive of acute leptospiral infection, it may not distinguish dogs with acute infection from those with chronic renal carriage of leptospires associated with other underlying diseases causing clinical manifestations similar to leptospirosis. Instead, the detection of leptospiral DNA from blood and serum samples must be considered a more reliable strategy to diagnose acute leptospirosis in dogs by molecular methods. Our results have also shown no distinction in laboratory findings and clinical outcome between confirmed and non-confirmed cases of leptospirosis, highlighting the non-specificity of clinical and laboratory evaluations for the diagnosis of the disease.

Both isolated strains could be characterized as *L. interrogans* serogroup Icterohaemorrhagiae and were shown to be pathogenic in a hamster model. The overall serological pattern found in the population studied indicates that the suspected dogs admitted to the veterinary hospital of the University of São Paulo, an ambulatory service that provides care for dogs from different regions of São Paulo city, are highly exposed to the Icterohaemorrhagiae serogroup. The isolation of serogroup Icterohaemorrhagiae from dogs with leptospirosis has been previously described in Brazil [21, 23], and previous serological surveys have consistently shown Icterohaemorrhagiae as the most reactive serogroup in dogs suspected of leptospirosis [15, 24–27]. Interestingly, the serological profile of human subjects suspected of leptospiral infection also indicates Icterohaemorrhagiae/Copenhageni as the main causative agents attributed to human infection [28–31], and most strains recovered from human patients with leptospirosis in Brazil were characterized as Icterohaemorrhagiae [29, 32]. Our findings corroborate the hypothesis that both dogs and humans are exposed to environmental contamination promoted by rodents [33], notably the brown rat (*Rattus norvegicus*), which was implicated as the main reservoir host of Icterohaemorrhagiae/Copenhageni serovars in urban areas from Brazil and other locations [34, 35].

Serological findings also revealed that leptospiral strains belonging to the Sejroe serogroup might be associated with acute leptospiral infection in dogs. This serogroup has been previously described as a causative agent of canine leptospirosis [36], and it was recently recovered from an asymptomatic dog in São Paulo city [12], thus indicating that Sejroe strains are actually circulating among dog populations in Brazil. In contrast, MAT titration against Canicola was only found in two dogs, which had low titers against this serogroup. Canicola strains are frequently isolated from dogs in Brazil and other locations [15, 16, 37], although serological evidence of Canicola infection is not often observed [38]. A previous study including dogs suspected of clinical leptospirosis conducted by our group between 2008 and 2012 found that even though most dogs reacted against serogroup Icterohaemorrhagiae, Canicola strains could still be recovered from three symptomatic dogs which had no MAT titers against this serovar [15]. All sequences from PCR-positive dogs could be identified as *L. interrogans* in this study, yet molecular characterization using single-gene sequencing methods has poor discriminatory power to distinguish among leptospires at a serovar/serogroup level [39], and infection caused by Canicola, although possible, could not be confirmed.

## Conclusions

Our results suggest that the use of multiple tests is essential for a more accurate and sensitive diagnosis of acute leptospirosis in dogs in a clinical setting. The results also suggest that despite the rising incidence of new serovars affecting dogs in different locations [3], mostly attributed to the extensive use of multivalent vaccines containing few representative serovars, and the increased contact between dogs and wildlife from rural-urban interface regions [5, 40, 41], the Icterohaemorrhagiae serogroup is still a major causative agent of canine leptospirosis in São Paulo, Brazil. Proper characterization of leptospiral isolates remain a crucial bottleneck to access the role of particular *Leptospira* strains in the epidemiology of canine leptospirosis and may provide evidence-based knowledge to support the development and commercialization of multivalent vaccines containing serovars that are circulating among local populations.

## Additional files

Additional file 1: Supplementary data from seven dogs revaluated after presenting clinical suspicion of leptospirosis. Additional file 1 shows data regarding immunization records, PCR, culture, DNA sequencing, MAT results and laboratory findings from the seven dogs that could be revaluated after presenting clinical suspicion of leptospirosis. (DOCX 144 kb)

Additional file 2: Supplementary data regarding clinical and laboratorial tests performed in the evaluation of the 33 suspected dogs included in the study. Additional file 1 shows confirmatory results from MAT, PCR and culture tests, as well as clinical outcome and clinical/laboratorial findings found in the first evaluation of each of the 33 suspected dogs included in the study. (DOCX 158 kb)

## Abbreviations

ALP: Alkaline phosphatase
ALT: Alanine aminotransferase
BUN: Blood urea nitrogen
CR: Creatinine
EDTA: Ethylenediamine tetraacetic acid
Ht: Hematocrit
MAT: Microscopic agglutination test
MLST: Multilocus Sequence Typing
PCR: Polymerase chain reaction
WBC: White blood cell
