# Supplementary material for: Diagnosis of acute canine leptospirosis using multiple laboratory tests and characterization of the isolated strains
Source: BMC Vet Res. 2018 Jul 17;14:222. doi: 10.1186/s12917-018-1547-4 (PMC6050646; doi:10.1186/s12917-018-1547-4)
Supplement: Supplementary file 1 — Supplementary data from seven dogs revaluated after presenting clinical suspicion of leptospirosis. Additional file 1 shows data regarding immunization records, PCR, culture, DNA sequencing, MAT results and laboratory findings from the seven dogs that could be revaluated after presenting clinical suspicion of leptospirosis. (DOCX 144 kb) [file 12917_2018_1547_MOESM1_ESM.docx]

**Additional file 1:** Supplementary data from seven dogs revaluated after presenting clinical suspicion of leptospirosis.

| Dog ID | Evaluation  Date | PCR | | DNA Sequencing | Culture | Vaccination | MAT titration (serovars) | | | | | | | | BUN | Creat | ALT | ALP | Ht | WBC |
| --- | --- | --- | --- | --- | --- | --- | --- | --- | --- | --- | --- | --- | --- | --- | --- | --- | --- | --- | --- | --- |
|  |  | Blood | Urine |  |  | (<1 year) | Po | Cas | Co | Ic | Br | Bu | Sh | Aut | mg/dL | mg/dL | IU/L | IU/L | % | 10^3^/mm^3^ |
| 14 | 06/30/14 | (-) | (-) | NP | (-) | Yes | 100 | - | - | - | - | - | - | 100 | 384.5 | 1.6 | 119.9 | 315.9 | 33.29 | 5 720 |
|  | 07/14/14 | NP | NP | NP | NP |  | 100 | - | - | - | - | - | - | 100 | 193.4 | 3.2 | 97.1 | 386.7 | 31.1 | 7 700 |
|  | 07/16/14 | NP | NP | NP | NP |  | - | - | - | - | - | - | - | - | 318 | 4 | 111.4 | 483.7 | 17.7 | 7 300 |
|  | 07/29/14 | NP | NP | NP | NP |  | 100 | - | - | - | - | - | - | - |  |  |  |  |  |  |
| 15 | 07/29/14 | (-) | (+) | (-) | (-) | No | - | - | - | - | - | - | - | - | 342.8 | 1.5 | 167.8 | 349.3 | 55 | 21 100 |
|  | 07/31/14 | NP | NP | NP | NP |  | - | - | 200 | - | 100 | - | 100 | - | 92.4 | 0.26 | 138.7 | 226.2 | 48 | 20 600 |
|  | 08/05/14 | NP | NP | NP | NP |  | - | - | 200 | - | - | - | 100 | - |  |  |  |  |  |  |
|  | 08/19/14 | NP | NP | NP | NP |  | - | - | - | 400 | - | - | - | - |  |  |  |  |  |  |
|  | 09/05/14 | NP | NP | NP | NP |  | - | - | - | 200 | - | - | - | - |  |  |  |  |  |  |
| 17 | 09/02/14 | (+) | (+) | (-) | (-) | No | - | - | - | - | - | - | - | - | 357.4 | 8.71 | 155.3 | 28.8 | 56.21 | 10 700 |
|  | 09/08/14 | NP | NP | NP | NP |  | - | - | - | - | - | - | - | - | 264.2 | 5.2 | 155.3 | 28.8 | 55 | 13 150 |
|  | 09/10/14 | NP | NP | NP | NP |  | - | - | - | - | - | - | - | - | 183.5 | 4 | 140 | 26 | 49 | 9 250 |
|  | 09/24/14 | NP | NP | NP | NP |  | - | - | - | - | - | - | - | - |  |  |  |  |  |  |
|  | 10/08/14 | NP | NP | NP | NP |  | - | - | - | - | - | - | - | - | 202.5 | 3.1 | 26 | 29.1 | 37.6 | 19 003 |
| 22 | 02/03/15 | (-) | (+) | *L. interrogans* | (-) | No | - | - | - | - | - | - | - | - | 434.3 | 6 | 24.1 | 27.9 | 30 | 18 940 |
|  | 02/06/15 | NP | NP | NP | NP |  | - | - | - | - | - | - | - | - | 538 | 9.2 | 47 | 53.3 | 48 | 19 450 |
| 24 | 04/02/15 | (-) | (+) | *L. interrogans* | (+) | Yes | - | - | - | - | - | - | - | - | 168.2 | 1.02 | 136.3 | 732.1 | 49 | 30 200 |
|  | 04/06/15 | NP | NP | NP | NP |  | - | - | - | - | - | - | - | - | 107.9 | 0.6 | 87.5 | 810.3 | 40 | 24 100 |
|  | 04/10/15 | NP | NP | NP | NP |  | - | - | - | - | - | - | - | - | 79.1 | 0.9 | 63.9 | 1206 | 43 | 20 720 |
| 25 | 04/23/15 | (-) | (-) |  | (-) | No | - | - | - | - | - | - | - | - | 281.4 | 1.7 | 1399 | 455 | 28 | 12 500 |
|  | 04/28/15 | NP | NP | NP | NP |  | - | - | - | - | - | - | - | - | 18.2 | 0.79 | 398.1 | 414 | 21 | 27 700 |
| 30 | 08/13/15 | (-) | (-) |  | (-) | No | - | - | 200 | 200 | - | - | - | - | 176.2 | 4.94 | 104 | 443.3 | 33 | 22 300 |
|  | 08/17/15 | NP | NP | NP | NP |  | 100 | 100 | 800 | 800 | 200 | - | - | - | 64.2 | 0.6 | 64.6 | 361 | 42 | 23 200 |
|  | 08/21/15 | NP | NP | NP | NP |  | - | 100 | 400 | 400 | - | - | - | - | 62.8 | 1.05 | 80.7 | 233.9 | 35 | 22 300 |

NP: not performed; Po: Pomona; Cas: Castellonis; Co: Copenhageni; Ic: Icterohaemorrhagiae; Br: Bratislava; Bu: Butembo; She: Shermani; Aut: Autumnalis.
