# Supplementary material for: Diagnosis of acute canine leptospirosis using multiple laboratory tests and characterization of the isolated strains
Source: BMC Vet Res. 2018 Jul 17;14:222. doi: 10.1186/s12917-018-1547-4 (PMC6050646; doi:10.1186/s12917-018-1547-4)
Supplement: Supplementary file 2 — Supplementary data regarding clinical and laboratorial tests performed in the evaluation of the 33 suspected dogs included in the study. Additional file 1 shows confirmatory results from MAT, PCR and culture tests, as well as clinical outcome and clinical/laboratorial findings found in the first evaluation of each of the 33 suspected dogs included in the study. (DOCX 158 kb) [file 12917_2018_1547_MOESM2_ESM.docx]

**Additional file 2:** Supplementary data regarding clinical and laboratorial tests performed in the evaluation of the 33 suspected dogs included in the study

| Dog ID | PCR-Confirmed Cases | Culture-confirmed cases | MAT-confirmed cases | BUN  (mg/dL) | CR  (mg/dL) | ALT  (IU/L) | ALP  (IU/L) | Ht  (%) | WBC  (10^3^/mm^3^) | Fever | Hypothermia | Anorexia | Vomiting | Diarrhea | Dehydration | Jaundice | Bleeding Disorders | Adipsia  Oligodipsia  Anuria  Dysuria  Oliguria | Other signs | | Clinical outcome |
| --- | --- | --- | --- | --- | --- | --- | --- | --- | --- | --- | --- | --- | --- | --- | --- | --- | --- | --- | --- | --- | --- |
| 1 | + |  |  | 540.6 | 14.8 | 97 | 100.1 | 51 | 21 900 |  |  | + | + |  |  |  | + | + |  | No follow-up | |
| 2 | + |  |  | 892 | 10.6 | 19.9 | 19.5 | 35 | 27 700 |  | + | + | + |  | + |  |  |  |  | Euthanasia | |
| 3 | + |  | + | 619.8 | 13.6 | 345 | 245 | 37 | 4 700 |  |  | + | + |  | + |  |  |  | T.N. | No follow-up | |
| 4 | + |  | + | 588.1 | 9.1 | 98.7 | 216 | 44 | 6 100 |  | + | + | + |  | + | + | + |  |  | Survived | |
| 5 | + |  |  | 147.7 | 2.1 | 274.9 | 1815 | 35 | 32 300 |  |  | + | + |  |  | + |  |  |  | Survived | |
| 6 |  |  |  | 208.3 | 2.03 | 147.4 | 37.2 | 51 | 23 000 |  |  | + | + |  | + |  |  |  |  | No follow-up | |
| 7 |  |  |  | 282.7 | 0.32 | 29.1 | 130 | 35 | 13 600 |  |  | + | + |  |  |  |  |  |  | No follow-up | |
| 8 | + | + |  | 498.3 | 16.3 | 130.7 | 170.9 | 48 | 35 000 |  |  | + | + | + | + | + |  |  | polydipsia | Survived | |
| 9 |  |  |  | 581.4 | 4.6 | 131.5 | 224.7 | 29 | 23 600 |  | + | + | + | + | + | + |  |  |  | No follow-up | |
| 10 | + |  |  | 316.3 | 4.4 | 420.8 | 1223.6 | 44 | 20 480 |  | + | + | + |  | + | + |  |  |  | No follow-up | |
| 11 |  |  | + | 116.6 | 1.3 | 268.1 | 1645 | 36 | 31 000 |  | + | + |  |  |  | + |  |  | T.N. | Survived | |
| 12 | + |  |  | 501 | 8.6 | 106 | 431.7 | 33.6 | 26 900 |  |  | + | + | + |  | + |  |  | polydipsia | No follow-up | |
| 13 |  |  |  | 429.4 | 3.5 | 114 | 222 | 53.6 | 62 600 |  | + | + | + |  |  |  |  | + |  | Died | |
| 14 |  |  |  | 384.5 | 1.6 | 119.9 | 315.9 | 33.2 | 5 720 |  |  | + |  | + |  | + |  |  |  | Died | |
| 15 | NR |  | + | 342.8 | 1.5 | 167.8 | 349.3 | 55 | 21 100 |  |  | + | + |  |  | + |  |  |  | Survived | |
| 16 | + |  |  | 792 | 21.6 | 186.8 | 376.1 | 28 | 17 100 |  | + | + |  | + |  | + |  | + | ataxia | Euthanasia | |
| 17 | NR |  |  | 357.4 | 8.71 | 155.3 | 28.8 | 56.2 | 10 700 |  |  | + |  |  |  |  |  |  |  | Survived | |
| 18 |  |  |  | 639 | 7.13 | 98 | 239 | 48 | 14 800 |  | + | + | + |  |  |  |  |  | convulsion | Survived | |
| 19 |  |  |  | 524.5 | 11.1 | 19.9 | 20.2 | 29 | 16 500 |  | + | + | + | + |  |  | + |  |  | Euthanasia | |
| 20 | NR |  |  | 576 | 10.9 | 206 | 1221 | 40 | 34 000 |  | + | + |  | + | + | + |  | + |  | Euthanasia | |
| 21 |  |  |  | 616.4 | 8.8 | 13 | 19.5 | 20 | 10 200 |  | + | + | + |  |  |  |  |  | Gingivitis | No follow-up | |
| 22 | + |  |  | 434.3 | 6 | 24.1 | 27.9 | 30 | 18 940 |  | + | + |  |  |  |  |  |  | polydipsia | Euthanasia | |
| 23 |  |  |  | 575.4 | 8.2 | 155.7 | 605 | 51 | 26 600 |  | + | + | + |  |  | + |  | + |  | No follow-up | |
| 24 | + | + |  | 168.2 | 1.02 | 136.3 | 732.1 | 49 | 30 200 |  |  | + |  |  |  | + | + |  | polydipsia | No follow-up | |
| 25 |  |  |  | 281.4 | 1.7 | 1399 | 455 | 28 | 12 500 |  |  | + | + |  | + | + |  | + | weakness | No follow-up | |
| 26 | NR |  |  | 439.8 | 4.27 | 35.2 | 409.2 | 24 | 14 400 |  | + | + | + | + |  | + | + |  |  | Died | |
| 27 |  |  |  | 488.8 | 9.7 | 73.3 | 717 | 28 | 38 000 |  | + |  | + |  |  | + |  | + |  | Euthanasia | |
| 28 |  |  |  | 259.8 | 3.35 | 26.1 | 35.5 | 33 | 5 030 |  |  | + |  |  |  |  |  |  |  | Survived | |
| 29 |  |  | + | 339 | 5.39 | 123 | 342 | ... | 20 000 |  |  | + | + | + | + | + | + | + |  | Euthanasia | |
| 30 |  |  | + | 176.2 | 4.94 | 104 | 443.3 | 33 | 22 300 |  |  | + | + |  |  | + | + |  |  | Survived | |
| 31 | + |  | + | 413.6 | 9.93 | 111.3 | 109.5 | 59 | 30 600 |  |  | + | + | + | + |  |  |  |  | No follow-up | |
| 32 | + |  | + | 378.4 | 8.9 | 114.5 | 397 | 27 | 38 900 |  |  | + | + | + |  | + |  | + |  | Survived | |
| 33 | + |  |  | 199.2 | 3.84 | 15.3 | 231.7 | 30 | 8 010 |  |  | + | + |  |  |  |  |  |  | No follow-up | |

T.N: Tongue necrosis; NR: Not readable
